# The Week's Work

**Published:** 1910-06-25

**Authors:** 


### The Week's Work.

### NORTH STAFFORDSHIRE INFIRMARY.

Mr. Spanton and his committee have done most excellent work in connection with this infirmary by their prompt action and the energy and ability they have displayed. It may be considered certain—thanks largely to them—that the North Staffordshire Infirmary buildings will in due course be brought up to date, and that this part of the hospital will so attain to the modern standard. The new out-patient department will no doubt prove a great blessing to the multitude of poor people who are mainly dependent upon its aid when ill. So far well. It is remarkable, however, that nothing but delay appears to result from every attempt to place the medical staff upon an adequate basis. One of the strongest points made in Sir Henry Burdett's report was the necessity for an increase in the staff. Yet Dr. Hind, speaking at Newcastle on the 10th inst., sneers at the report which has resulted in such momentous changes, and goes the length of stating that Sir Henry had not a word to say with regard to the in-patients. It is perfectly clear that, if the needs of the in-patients are to be properly met on medical and surgical grounds, there must be an adequate medical staff, and so long as it remains inadequate the in-patients cannot possibly have all the attention which they would otherwise receive. The truth is that everyone who has an intimate knowledge of the internal working of the North Staffordshire Infirmary knows that there are certain members of the staff who look with grave disfavour upon the proposal to increase the staff. We may properly point out to Dr. Hind that the paramount claims of the in-patients make such action unworthy of the example set by the leading members of the medical profession, who place efficiency in hospital work before personal considerations of every kind. King Edward set a splendid example by his consideration for the interests of the humblest of his subjects, and we venture to hope that no personal considerations will be permitted to delay longer the increase in the medical staff of the North Staffordshire Infirmary, which is urgently called for, and can, and ought to be promptly made. Probably Sir Henry took it for granted that the medical staff would gladly co-operate with the lay committees in doing everything possible to further the best interests of the in-patients. Be this as it may, some one should promptly make it his business to see that further delay in making the new medical appointments which are so urgently called for is made impossible. The demand for the reorganisation of the medical staff is urgent, and it should not be longer postponed, no matter who may attempt to obstruct these reforms, or what may be the motives which underlie such obstruction.

### WHO BENEFITS?

Our attention has been called to four advertisements from different institutions which appeared in the morning papers, each acknowledging a £5 bank-note from an appeal in a weekly newspaper. These advertisements, we are informed, appeared on three successive days, and the actual cost to these four institutions could not have been less than three guineas for each newspaper in which they appeared, so that the expense to the charities for advertising must have absorbed the larger portion of the so-called gifts. We call attention to the matter because it marks a new departure in journalism which cannot prove of advantage to the charities, however advantageous it may be to the proprietors of a newspaper which adopts this extraordinary method of dealing with small sums placed in their hands for that purpose. It has been suggested to us that the proprietors of the weekly newspaper in question paid for these advertisements themselves as an attractive method of increasing the number of advertisements which appear in their columns. This we cannot credit, but should it be true we may point out that hospital secretaries are not quite such innocent people as the obvious inference would imply.

### HOSPITAL GRANTS FROM THE CITY CORPORATION.

It is announced that an attempt will be made to raise a discussion on the appointment of a new matron of St. Bartholomew's Hospital at the Court of Common Council this week, after we go to press. We cannot understand the grounds upon which this most undesirable departure from precedent is to be attempted, and we hope that the Lord Mayor may rule the discussion out of order. The Court of Common Council is adequately represented on the governing body of St. Bartholomew's Hospital, and if there is any matter which interests the Council it should be raised in regular and proper form through one of their representatives in the ordinary way. A correspondent calls our attention to the extraordinary questions which are sometimes asked hospital deputations who apply to the Common Council at the Guildhall for a grant. On a recent occasion the first question asked was, "Why do you (a hospital spending £40,000 a year) give your secretary £450 a year?" It does not appear whether this question was asked with the object of enforcing on the governors the fact that the salary was too small for the responsibilities of the office, but we think that is possible, as it would certainly have been justifiable, a point which our correspondent seems to have missed. The questioners could certainly not have imagined that the secretary was overpaid in the circumstances. We have always maintained, and experience proves we are right, that it is false economy to underpay hospital workers, and especially those who are responsible for raising an adequate income to maintain a hospital in a state of the highest efficiency. Three per cent. upon the total income is not too high a sum to pay a really efficient chief officer of a great voluntary hospital. Of late years several men of university training and high ability have become secretaries of voluntary hospitals. If this practice is to continue, as we hope it may do, it is perfectly certain that these gentlemen must be offered emoluments at least equal to the average sum which they could have earned had they entered one of the learned professions.

### MEDICAL SOCIOLOGY.

A good deal of interest should centre round the discussions which are being organised at the annual meeting of the British Medical Association to be held in London at the end of July. Papers will be read on the Economical Basis of Hospital Management. Section 1 includes the provision of funds from three sources: (*a*) public grants, (*b*) charitable or philanthropic gifts, (*c*) payments by patients. Section 2 will deal with the conditions of admission of in- and out-patients, including the work of an almoner. The economical basis of the out-patient department is to be considered from the professional, the patient's, and the hospital point of view. The treatment of patients by dispensaries, and also in consultation departments of the hospitals, will be dealt with. We hope that those who know most about the subjects mentioned will endeavour to make it their business to attend this meeting and to take part in the discussions. There can be no doubt that the present offers a unique opportunity to hospital managers, the medical profession, municipalities, public health departments, and poor-law administrators to unite in the formulation of a plan whereby, through co-operation, Great Britain may have a medical service suitable to the needs of all classes of the community, whilst it safeguards every professional interest. Once this co-operation is secured we should possess in practice the most perfect service for the sick in the world.

### PRACTICAL PHILANTHROPY IN EDINBURGH.

Two experiments in hospital management are in process of evolution in Edinburgh, to both of which we very cordially wish success, for both are attempts to provide help for those who are able and willing to help themselves to the extent that their means allow. Both schemes, in fact, are aimed at the relief of that large class of the public whereon the burden of an expensive operation in a nursing home run for profit is one which is absolutely crushing, whereas the advantage of free hospital treatment is either refused by the almoner or repellent to the individual's self-respect. The first of these is a hospital for women, which will commence with accommodation for eighteen in-patients and with two surgeons, and to which three assistant-surgeons are also appointed to deal with out-patients. The committee of management has raised sufficient funds for the purchase of a pre-existing home, and has drawn up a series of rules which are to be strictly adhered to. Thus no one is to be admitted, either as an in-patient or an out-patient, except on the recommendation of her general practitioner, who is to be the sole judge of her fitness for the benefits of the hospital. A uniform charge of £1 per week is to be paid by all in-patients for maintenance; but the services of the medical staff are, of course, to be purely honorary and unremunerated. The other scheme, which has not progressed so far towards completeness as the former, is in essence a nursing home promoted and managed by the medical profession. The promoters aim at raising £10,000 with which to provide, equip, and maintain a nursing home for the lower, middle, and upper working classes. The rates charged are to vary from 10s. 6d. to £3 3s. a week, according to the means of the patients. There will be no visiting staff; patients will select their own medical man, who will be remunerated by the patient as if the latter were being treated at his or her own home. In commenting on this proposal the *Edinburgh Medical Journal* very wisely suggests a regulation that patients who pay the minimum for admission should be charged also the lowest possible medical fees. This is recommended, not on the ground that any such proviso is really necessary—we entirely agree that it is not—but simply that the charitable public, to whom an appeal for part of the £10,000 is to be issued, may have no shadow of reason to doubt the disinterestedness of the experiment as far as the medical profession is concerned. When once the home is started it should require no charitable aid; but it is to be hoped that the promoters will not commence work until they are assured of sufficient funds to start it with a thoroughly adequate building and equipment.

### THE CERTIFICATION OF AN INSTITUTION.

Judging from the contents of our letter-box, considerable uncertainty seems to prevail as to the course to be followed for securing the certification of an institution by the Local Government Board. The procedure for securing the certification of an institution under the Poor Laws, etc., Act of 1862 (25 & 26 Vict. chap. 43) is merely to apply to the Local Government Board and support the application with as full information as possible respecting the institution, its aims, accommodation, management, etc. If the Board regard the institution as coming within the Act, it will then be visited and reported on by one of the Board's inspectors, and his report to the Board will decide as to certifying. There is no expense connected with certification by the Board as such, but, of course, the Board might find it necessary to attach as conditions of certification certain alterations or extensions of buildings.

### THE DRUG MARKET.

Business has been quiet and uninteresting during the week, and few price changes of any note have taken place. Up to the present the expected advance in the price of bromide salts has not been announced, and in the meantime makers are accepting orders for small quantities at the old prices, but will not book orders for forward delivery. American peppermint oil is rather lower owing to favourable reports of the growing crop. At the recent public drug sales Cape aloes fetched slightly higher rates, coca leaves tended dearer, and guaiacum was also rather dearer, otherwise there were practically no changes in price.

